# The effects of hypothermia on glutamate and γ-aminobutyric acid metabolism during ischemia in monkeys: a repeated-measures ANOVA study

**DOI:** 10.1038/s41598-022-18783-8

**Published:** 2022-08-25

**Authors:** Bo-hu Liu, Jun Pu, Ze-qi Li, Xiao-ran Zhang

**Affiliations:** 1 The Second Affiliated Hospital of Kunming Medical University, Kunming, 650101 Yunnan China; 2 Calmette Hospital Affiliated to Kunming Medical University, Kunming, 650032 Yunnan China

**Keywords:** Hypoxic-ischaemic encephalopathy, Stroke

## Abstract

During an ischemic stroke, the brain releases various factors, including glutamate and γ-aminobutyric acid. Glutamate can cause neurotoxic effects through certain receptors and exacerbate neurological damage, while γ-aminobutyric acid as an inhibitory neurotransmitter can antagonize the excitotoxic effects of glutamate and enhance the tolerance of neurons to ischemia. Therefore, in this study, the content of amino acid neurotransmitters in brain tissue before ischemia, after 10 min of ischemia, hypothermic perfusion, and rewarming were analyzed by high-performance liquid chromatography-UV in an animal model of ischemic stroke generated by blocking the bilateral common carotid arteries of rhesus monkeys. The changes in amino acid neurotransmitters in the rhesus monkey brain during post-ischemia hypothermic perfusion and rewarming were investigated by statistical methods of repeated measures ANOVA, showing that the concentration change of glutamate had not only a temporal factor but also was influenced by temperature, and there was an interaction effect between the two. Time but not temperature affected the change in γ-aminobutyric acid concentration, and there was an interaction effect between the two. Accordingly, hypoperfusion exerts a protective effect during ischemia by inhibiting the release of excitatory amino acid neurotransmitters, while the antagonistic effect of GABA on Glu is not significant.

## Introduction

Ischemic stroke is a major disease that endangers human health and life and is a major cause of death and long-term disability^1^. The acute damage of brain tissue ischemia usually occurs minutes to hours after ischemia, leading to a large release of glutamate from neurons and an increase in intracellular calcium ions, resulting in an "excitotoxic" effect. In turn, the cell membrane depolarizes, which further leads to a large calcium ion influx and glutamate release, forming a vicious circle, with water molecules also entering the cell in large quantities leading to cytotoxic edema^2,3^, thereby neuronal damage, destruction of cell membranes, nematodes, and DNA, as well as the stimulation of neuronal apoptosis, necrosis and neuroinflammatory responses^3^. Glutamate (Glu) is an excitatory amino acid, and neurons receive only small amounts of glutamate stimulation under normal conditions. However, during ischemia, the prominence of the presynaptic membrane is continuously depolarized, resulting in massive Glu release, excessive activation of Glu receptors on the postsynaptic membrane, and mediates excessive activation of NMDA receptors leading to cell death^4,5^. Its metabolite, γ-aminobutyric acid, is an inhibitory neurotransmitter that acts as its natural antagonist in extreme sensitivity to cerebral ischemia, often leading to Cl^–^ influx and generating inhibitory potentials in the postsynaptic and anterior membranes to rescue neuronal apoptosis^6,7^.

The effects of hypothermia treatment occur in different phases of ischemic stroke, which include affecting ion homeostasis as well as neurotransmitter release in the acute phase and delaying neuronal cell death due to a combination of apoptosis, inflammation, oxidative stress, and excitotoxic pathways in the subacute phase. Consequently, therapeutic hypothermia may affect almost every one of these pathways, and this multifaceted mechanism is believed to account for its powerful therapeutic effects^8^. In the present study, we established a monkey brain ischemia model to understand the molecular pathways of cells affected by hypothermia.

Repeated measurement data are indicators obtained from multiple measurements of the same observation on the same subject at different points in time. Analysis of variance (ANOVA) of repeated measurement data is usually used to analyze the characteristics of changes in the observed index at different time points^9^. In this experiment, the rhesus monkeys were blocked from the common carotid arteries bilaterally for 10 min at room temperature to simulate an animal model of ischemic stroke. The changes in amino acid neurotransmitter metabolism were observed before ischemia, within 10 min of ischemia, and during hypothermic perfusion and rewarming. ANOVA was used to observe the changes in amino acid neurotransmitters in the rhesus monkey brain after ischemia and rewarming to further understand the damage mechanism of neurons by ischemia and hypoxia from the perspective of transmitter metabolism and to explore the effect of hypothermic perfusion on the metabolism of amino acid neurotransmitters in rhesus monkey brain ischemia.

## Materials and methods

### Experimental materials

Nine healthy adult rhesus monkeys weighing 8.22 ± 2.15 kg aged 7.90 ± 1.82 years were used in this study that was conducted in strict accordance with the recommendations in the guidelines for the care and use of laboratory animals issued by the National Institutes of Health and the ARRIVE guidelines. The animal use agreement was reviewed and approved by the animal use Organization Committee of the Second Affiliated Hospital of Kunming Medical University.

### Establishment of the animal model and specimen collection and processing

**Figure 1:**
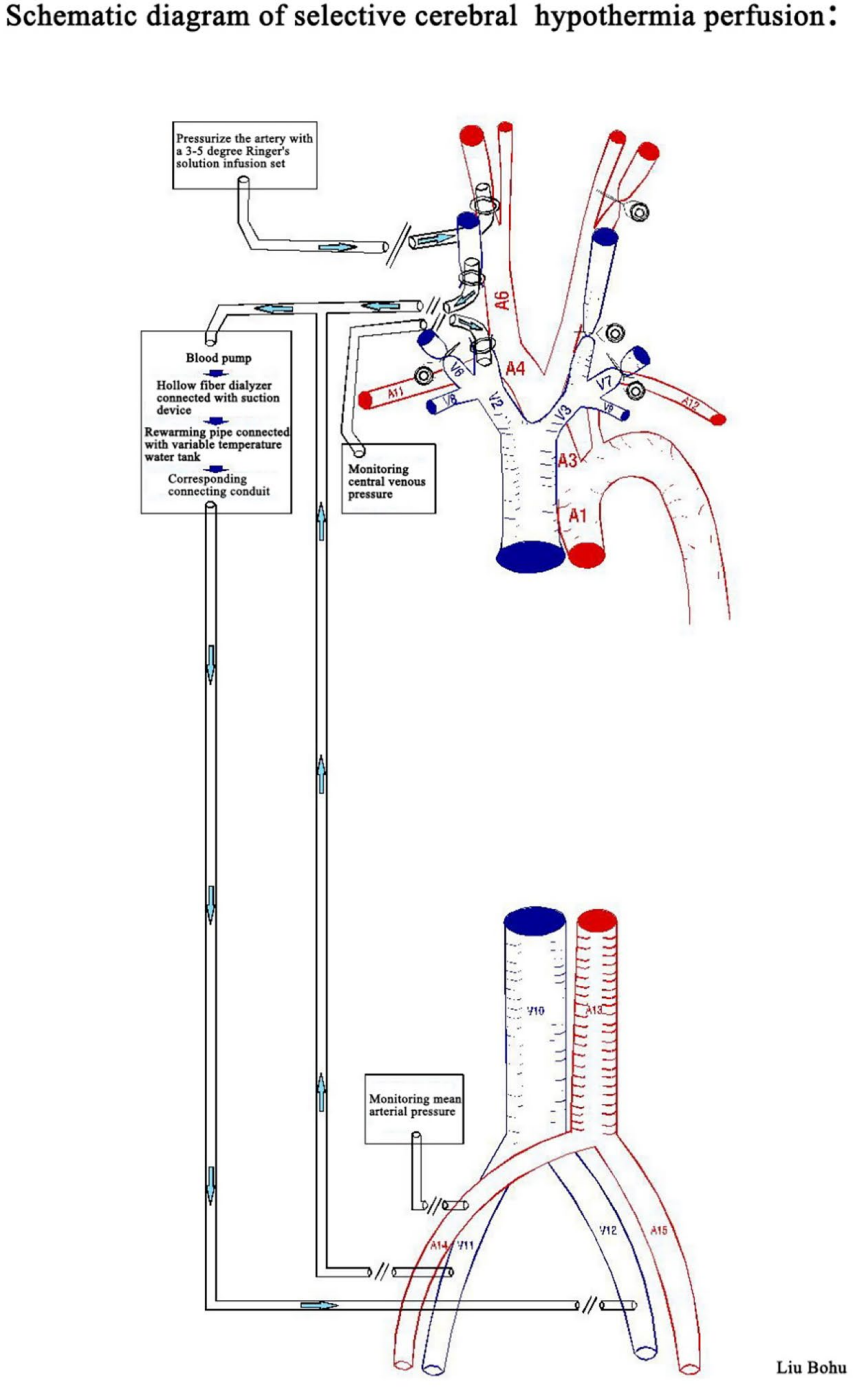
The map of selective cerebral deep hypothermic perfusion. A1 ascending aorta, A2 aortic arch, A3 common trunk, A4 cephalic trunk, A5 left common carotid artery, A6 right common carotid artery, A7 right internal carotid artery, A8 left internal carotid artery, A9 right external carotid artery, A10 left external carotid artery, A11 right subclavian artery, A12 left subclavian artery, A13 abdominal aorta, A14 right femoral artery, A15 left femoral artery, V1 superior vena cava, V2 right cephalic arm vein, V3 left cephalic arm vein, V4 right internal jugular vein, V5 left internal jugular vein, V6 right external jugular vein, V7 left external jugular vein, V8 right subclavian vein, V9 left subclavian vein, V10 inferior vena cava, V11 right femoral vein, V12 left femoral vein.

**Figure 2:**
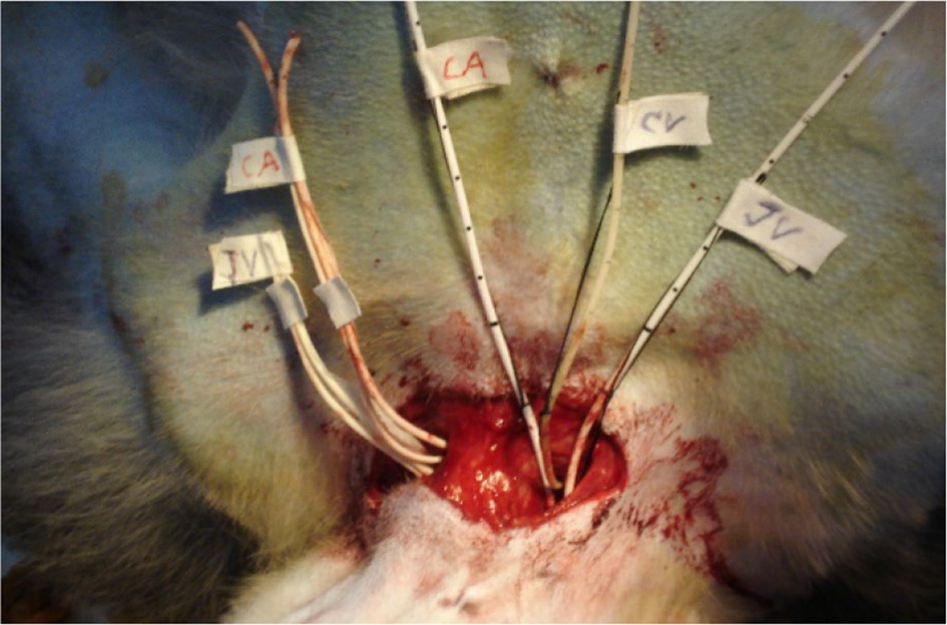
Separation of bilateral jugular arteries and placement of tubes.

**Figure 3:**
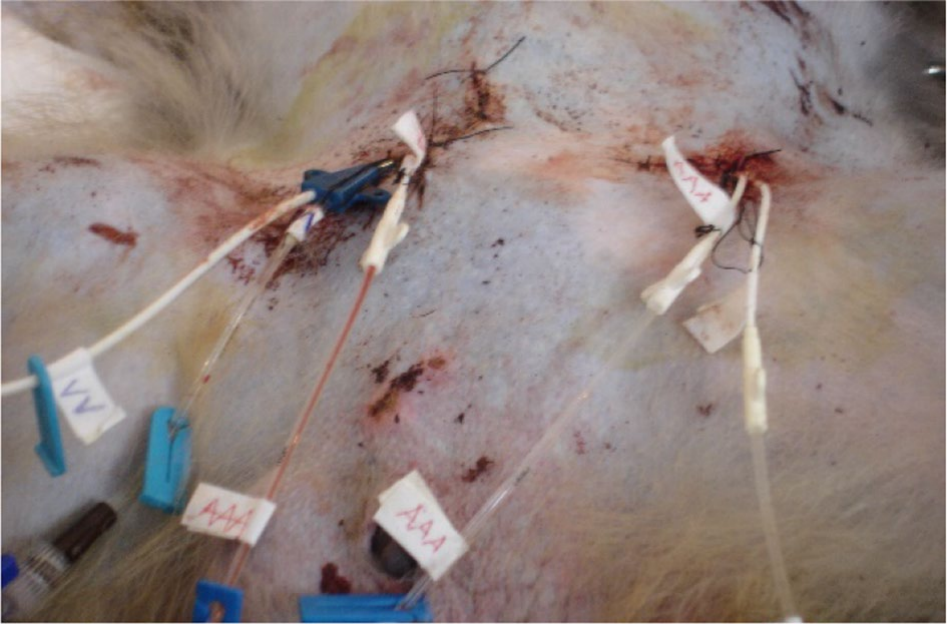
Separation of bilateral femoral arteries and placement of tubes.

**Figure 4:**
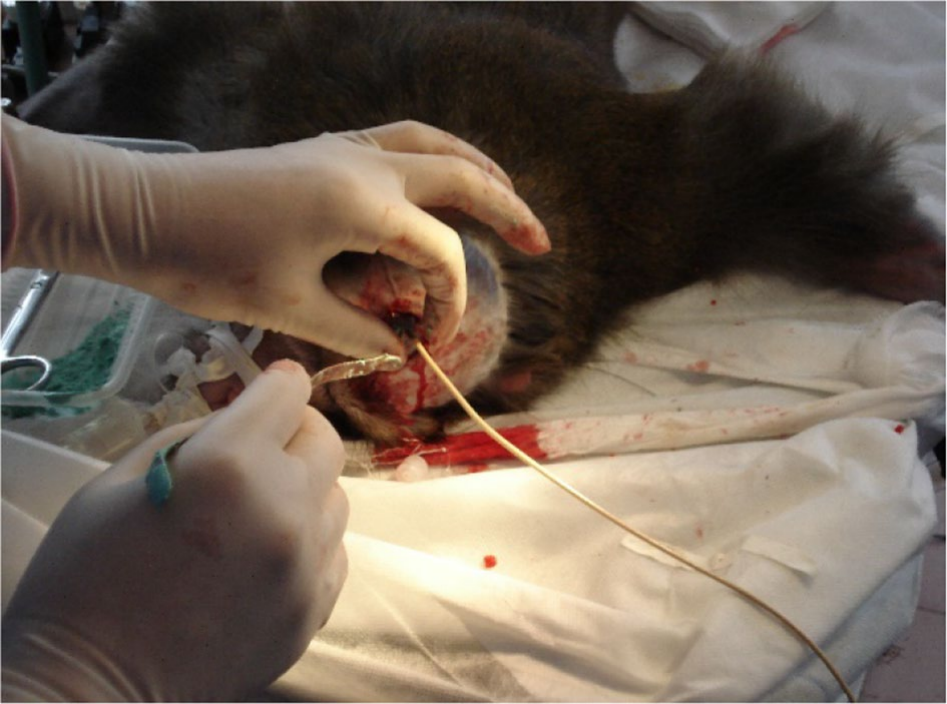
Drilling of the right frontal lobe and insertion of the brain temperature sensor.

**Figure 5:**
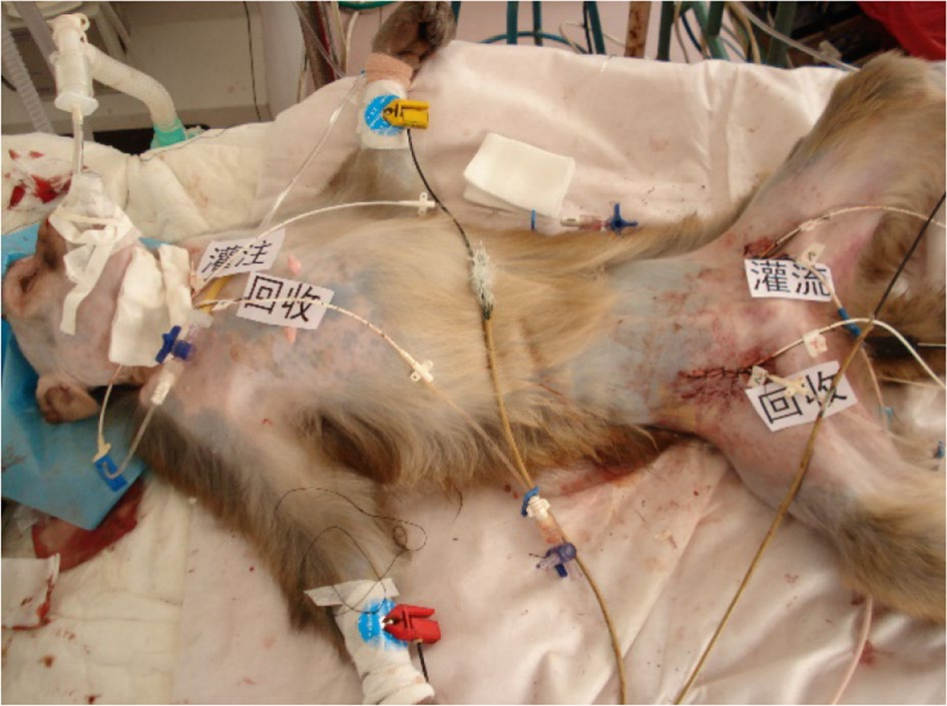
Schematic diagram of the hypothermic diversion vessels.

**Figure 6:**
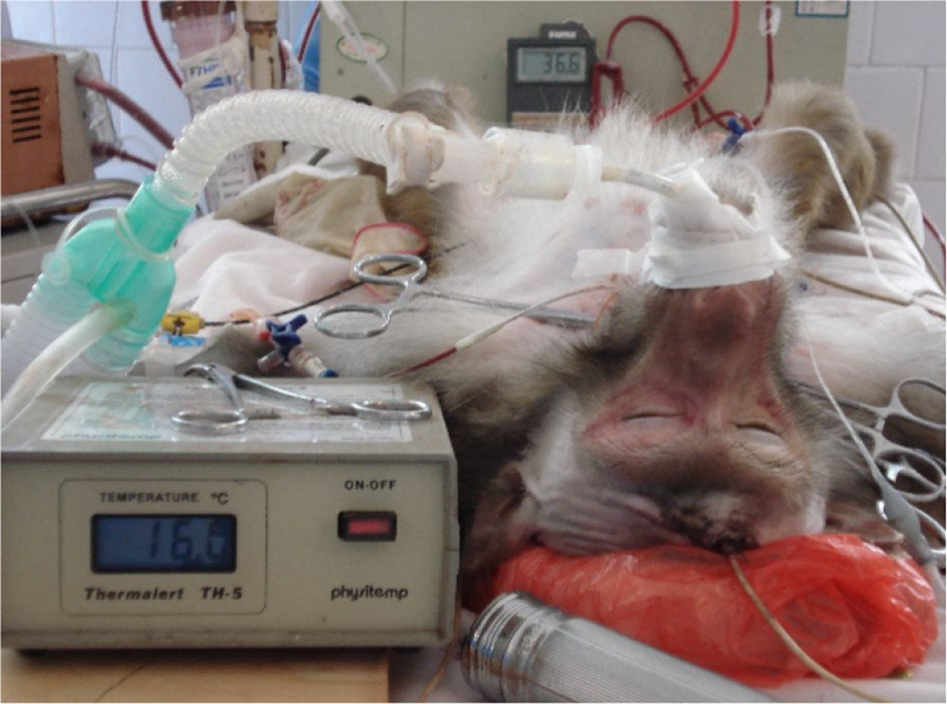
Brain temperature dropped below 18 °C.

**Figure 7:**
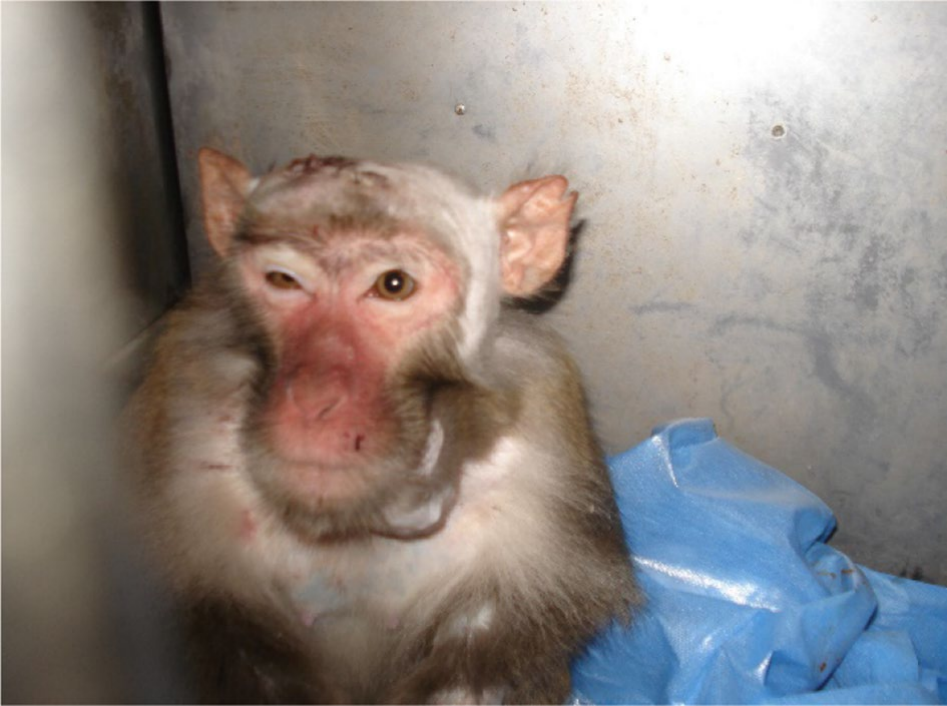
Experimental monkeys were successfully resuscitated.

The cerebral ischemia and hypothermic perfusion model was established according to the previous method^5^, as displayed in Fig. 1. Induction of anaesthesia was performed with ketamine hydrochloride (10 mg/kg) and diazepam (0.5 mg/kg), and the procedure was initiated after effect. Figures 2, 3, 4, 5, 6 and 7 show the procedure. After temporary blockade of the bilateral common carotid arteries for 10 min, 4.0 ± 0.5 °C perfusion solution was injected via the distal end of the right internal carotid artery, and excess water was removed by ultrafiltration, rewarmed to 37 °C, and returned to the body circulation system, which rapidly lowered the temperature of brain tissue to ≤ 18 °C. The low-temperature perfusion was maintained for 60 min, the temporary blockade was lifted, normal blood flow was restored, the experimental animals were naturally rewarmed, and the rewarming process lasted for 60 min. A microdialysis needle was placed in the right parietal lobe, and extracellular fluid was collected from the brain through the microdialysis device starting from 10 min before ischemia. One tube was collected every 20 min until 60 min of rewarming, including one tube before 10 min of ischemia and one tube during 10 min of ischemia, for a total of 8 tubes.

The specimens were analyzed qualitatively and quantitatively by high-performance liquid chromatography (HPLC)-UV to determine the concentrations of glutamate and γ-aminobutyric acid in the extracellular fluid of brain tissue (Figs. 8, 9, 10, 11, 12, 13, 14, 15).

**Figure 8:**
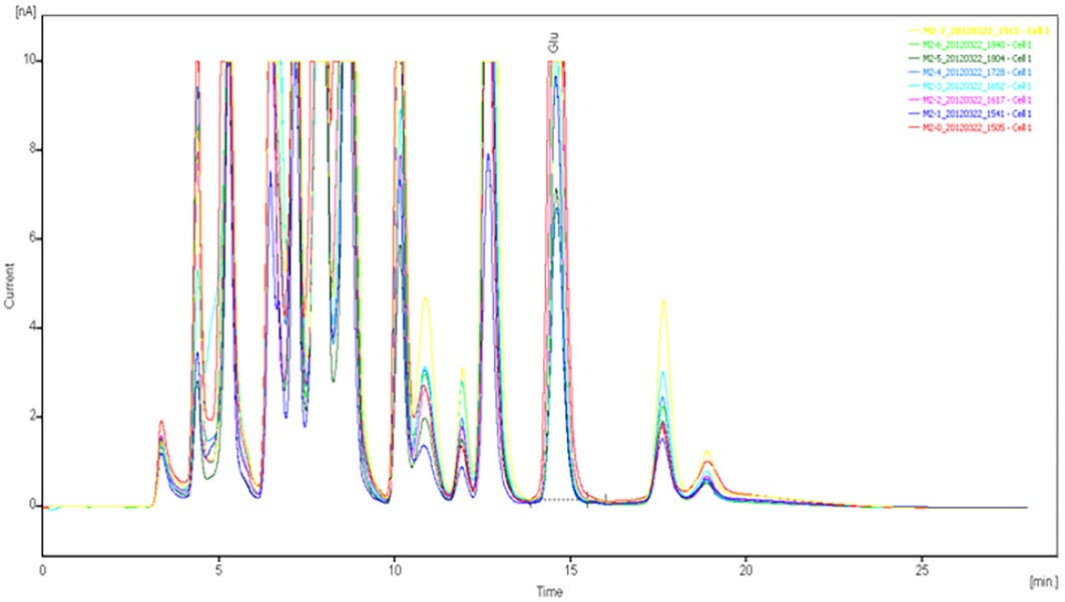
Glu high performance liquid chromatogram1.

**Figure 9:**
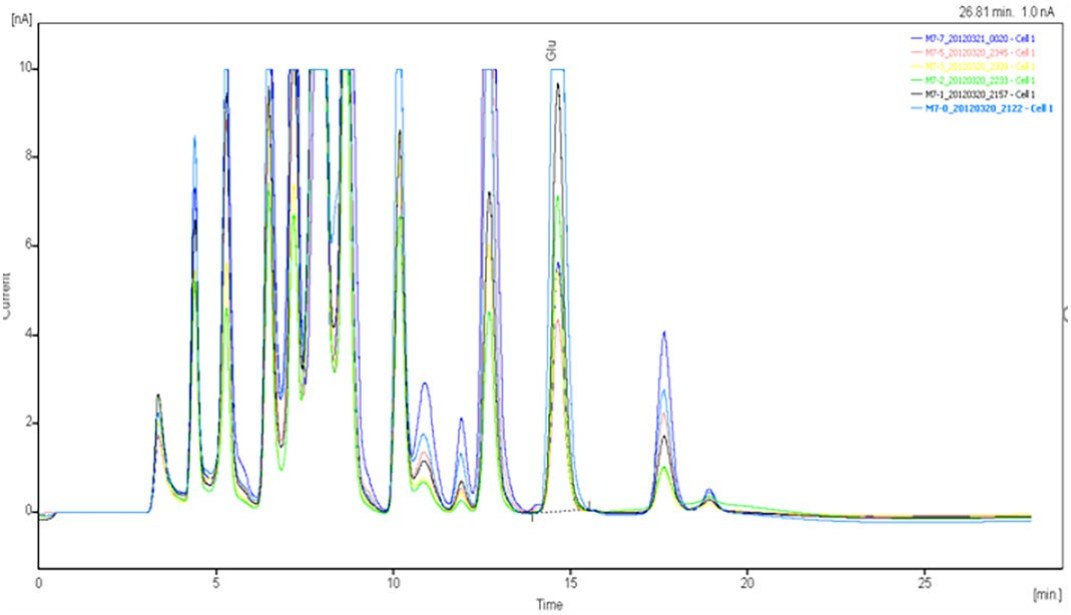
Glu high performance liquid chromatogram2.

**Figure 10:**
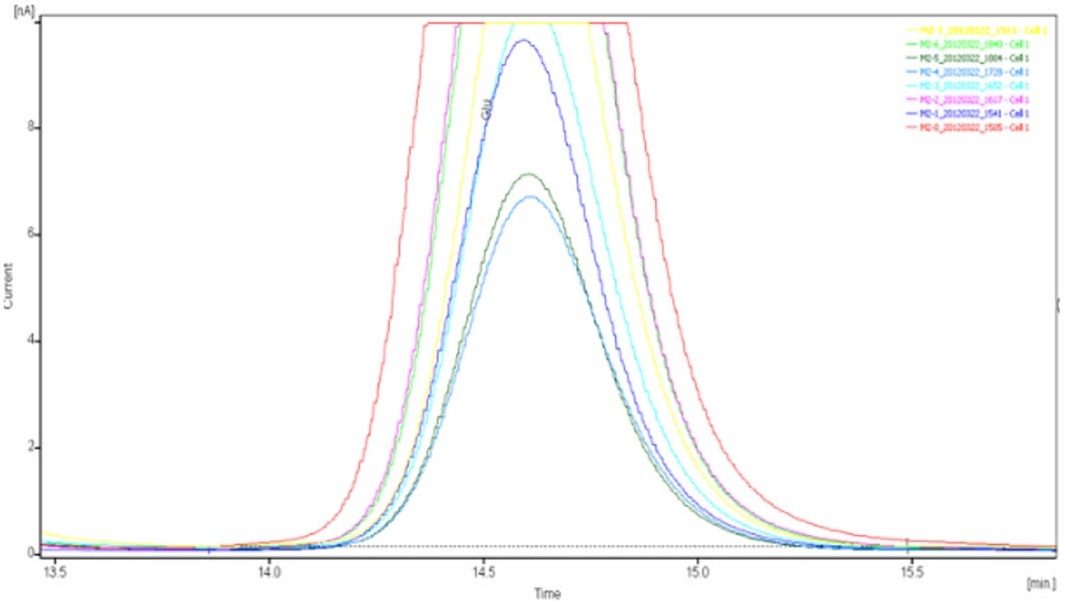
Glu high performance liquid chromatography3.

**Figure 11:**
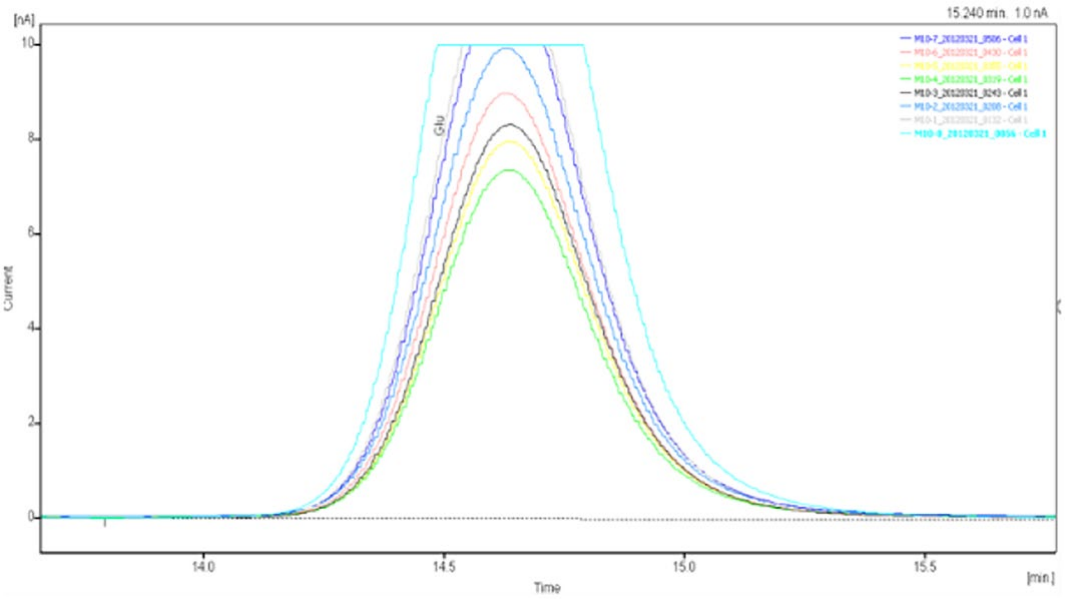
Glu high performance liquid chromatography4.

**Figure 12:**
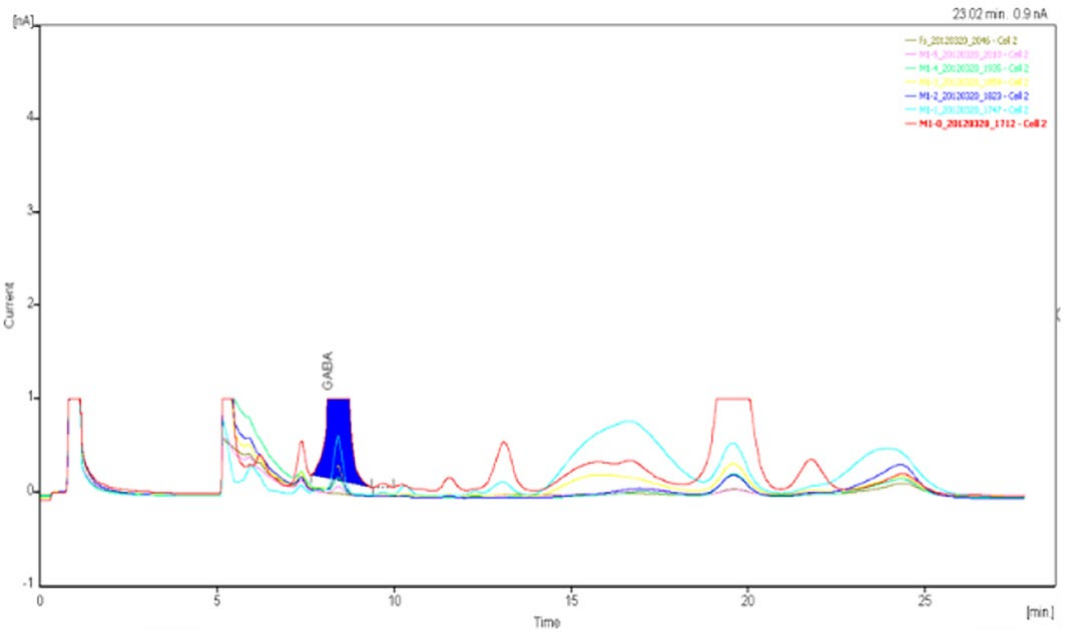
GABA high performance liquid chromatogram1.

**Figure 13:**
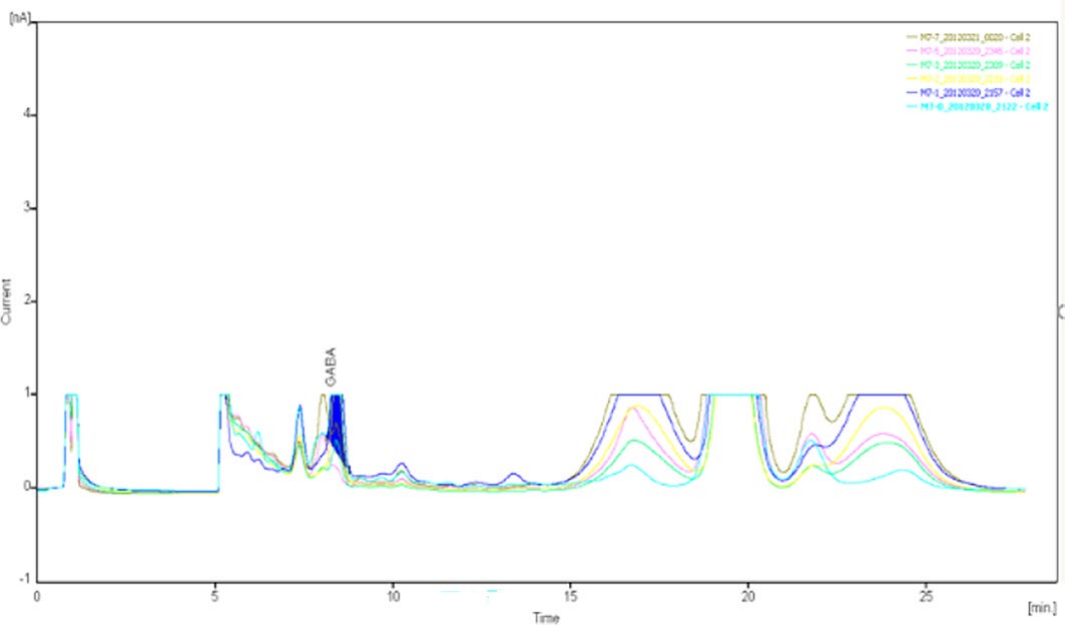
GABA high performance liquid chromatogram2.

**Figure 14:**
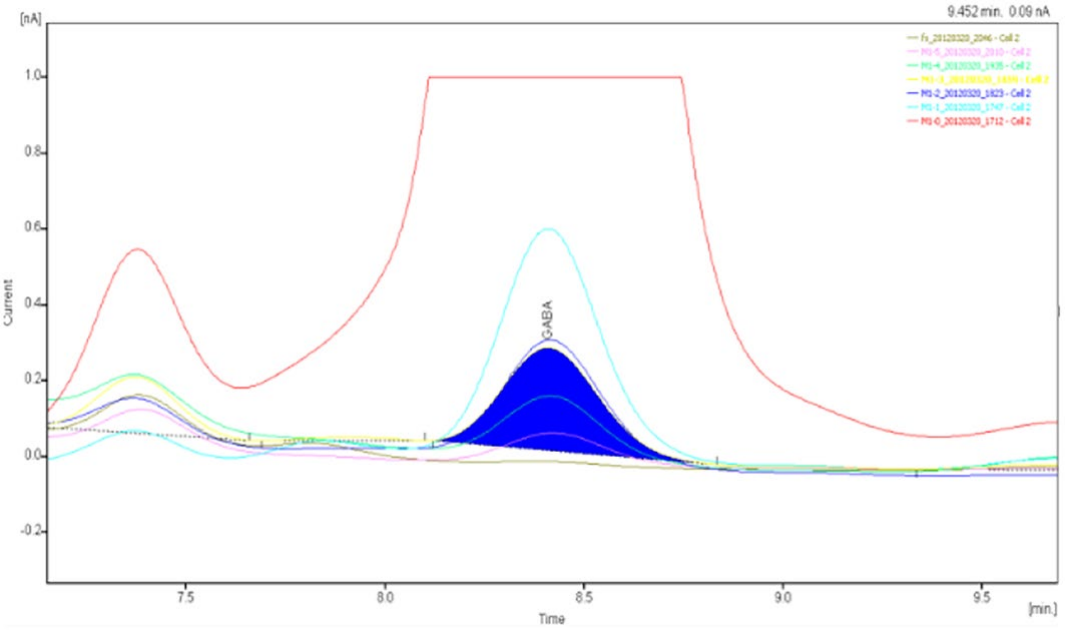
GABA high performance liquid chromatography3.

**Figure 15:**
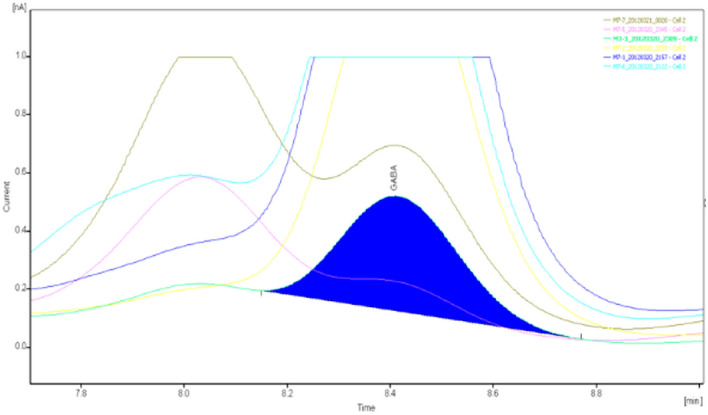
GABA high performance liquid chromatography4.

### Statistical analysis

SPSS 26.0 software was used for statistical analysis. The concentration of Glu and GABA in different groups was compared, and all quantitative data were expressed as mean ± standard deviation ($\overline{x}$ ± s). The data were tested for normality, a parametric test for normally distributed data, and a non-parametric test for non-normally distributed data. One-way ANOVA was used to compare the means of multiple groups that met the normal distribution with homogeneity of variance, and LSD method was used for a two-way comparison between means for those with differences. If the data did not meet the normal distribution, the rank-sum test (Kruskal–Wallis H test) was used to compare multiple independent samples in a completely randomized design. Glu and GABA concentrations were compared with two-time groups of temperature (hypothermic 20 min group, hypothermic 40 min group, hypothermic 60 min group, rewarmed 20 min group, rewarmed 40 min group, rewarmed 40 min group, rewarmed 20 min group). The Glu and GABA concentrations were measured at different time points for the same subject and were repeatedly measured. Since temperature and time may affect the observed data, two-factor repeated-measures ANOVA was applied for statistical analysis, and the sphericity test was performed before analysis. If the data did not satisfy the sphericity test, the Greenhouse–Geisser method was used for correction. The confidence interval of degrees of freedom was a = 0.05, and P < 0.05 was considered statistically different.

## Results

### Analysis of changes in Glu content in brain extracellular fluid

**Figure 16:**
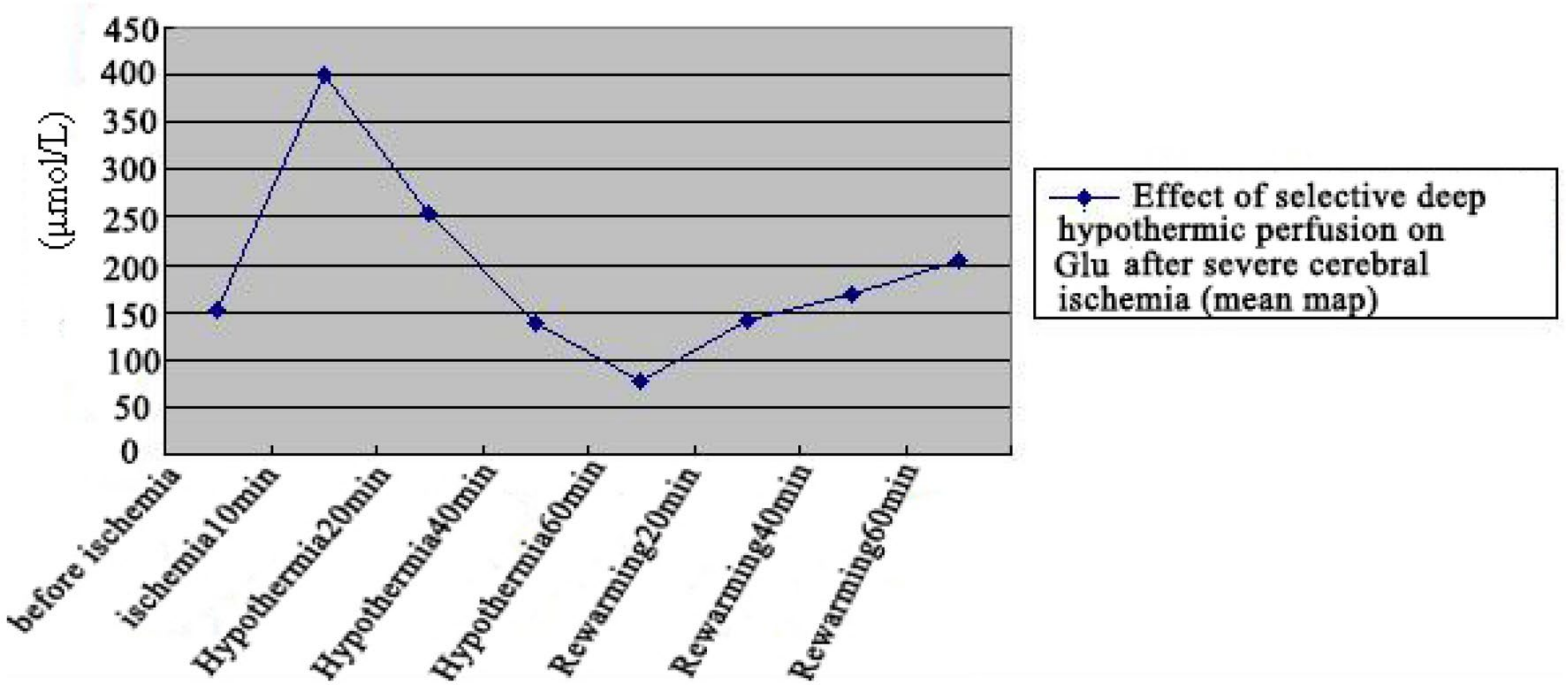
Changes of Glu content in brain extracellular fluid.

Glu concentration was determined in the extracellular fluid of brain tissue before 10 min of ischemia, after 10 min of ischemia, and during hypothermic perfusion and rewarming, and all quantitative data were expressed as mean ± standard deviation ($\overline{x}$ ± s) (Table 1). One-way ANOVA demonstrated significant Glu concentration differences between treatment groups (F = 279.66). There were significant differences in multiple comparisons between groups by the LSD method, and Glu concentration values in extracellular fluid reached the highest value at 10 min of ischemia (398.99 ± 34.03 µmol/L), which was significantly higher than the groups before ischemia, at different times of hypothermic perfusion and rewarming. There was a decreasing trend during 20, 40, and 60 min of hypothermic perfusion, and reached the lowest value (76.32 ± 8.08 µmol/L) at 60 min of hypothermic perfusion and showed an increasing trend during 20, 40, and 60 min of rewarming (Fig. 16).

**Table 1:** Changes in Glu content (μmol/L) in brain extracellular fluid (± s n = 9).

Timing	Glu
Before ischemia	150.81 ± 12.02*^▲^
Ischemia for 10 min	398.99 ± 34.03
Hypothermic 20 min	253.58 ± 19.31*^▲^
Hypothermic 40 min	137.20 ± 13.42*^▲^
Hypothermic 60 min	76.32 ± 8.08*
Rewarmed 20 min	169.11 ± 13.02*^▲^
Rewarmed 40 min	203.80 ± 15.46*^▲^
Rewarmed 60 min	191.37 ± 94.32*^▲^
F	279.66
P	0.00

*Compared with ischemia for 10 min, *P < 0.05.

^▲^Compared with rewarmed 60 min, ▲P < 0.05.

Spearman correlation analysis of Glu concentrations with time groups in two temperature zones (cryogenic 20 min group, cryogenic 40 min group, cryogenic 60 min group, rewarming 20 min group, rewarming min group, and rewarming min clock group) depicted that the Glu content was significantly negatively correlated with cryogenic perfusion time (rs = − 0.94**, P = 0.00) (Table 2) and significantly positively correlated with rewarming time (rs = −0.91**, P = 0.00) (Table 3).

**Table 2:** Glu values of brain extracellular fluid in different hypothermic perfusion time groups (μmol/L).

Timing	n	$\overline{x}$ ± s (μmol/L)	Rs	P
Hypothermic 20 min	9	253.58 ± 19.31	−0.94**	0.00
Hypothermic 40 min	9	137.20 ± 13.42		
Hypothermic 60 min	9	76.32 ± 8.08		

There was a significant correlation between different hypothermic perfusion times and Glu values.

**When the confidence (bilateral) is 0.01, the correlation is significant.

**Table 3:** Glu value of brain extracellular fluid in different rewarming time groups (μmol/L).

Timing	n	$\overline{x}$ ± s (μmol/L)	Rs	P
Rewarmed 20 min	9	141.14 ± 13.07	0.91**	P = 0.00
Rewarmed 40 min	9	169.11 ± 13.02		
Rewarmed 60 min	9	203.80 ± 15.46		

There was a significant correlation between different rewarmed times and Glu values.

**When the confidence (bilateral) is 0.01, the correlation is significant.

According to Mauchly sphericity test P < 0.10, the data do not conform to the spherical data. There was a correlation between the results of multiple measurements, so ANOVA of repeated measurements was used, and the results were corrected using the Greenhouse–Geisser method. The results of ANOVA of repeated measurements of Glu, F = 124.319 after correction of the internal factor, P < 0.10, indicated that the overall means of Glu at different times were not the same, while the interaction time × temperature corrected F = 502.868, P < 0.10, indicated that there was an interaction between temperature and treatment time and that temperature had an effect on the measurements. The overall mean of Glu was not the same for the two temperature patterns F = 9.797, P < 0.10 (Table 4).

**Table 4:** Analysis of variance of repeated measurement of Glu.

Variation	Sum of squares of deviation (SS)	Degrees of freedom (df)	Mean square	F	P
Time	32,459.595	1.566	20,731.524	124.319	0.000
Temperature	3306.908	1	3306.908	9.797	0.006
Time × temperature	131,298.32	1.566	83,858.541	502.868	0.000

### Analysis of GABA content in brain extracellular fluid

**Figure 17:**
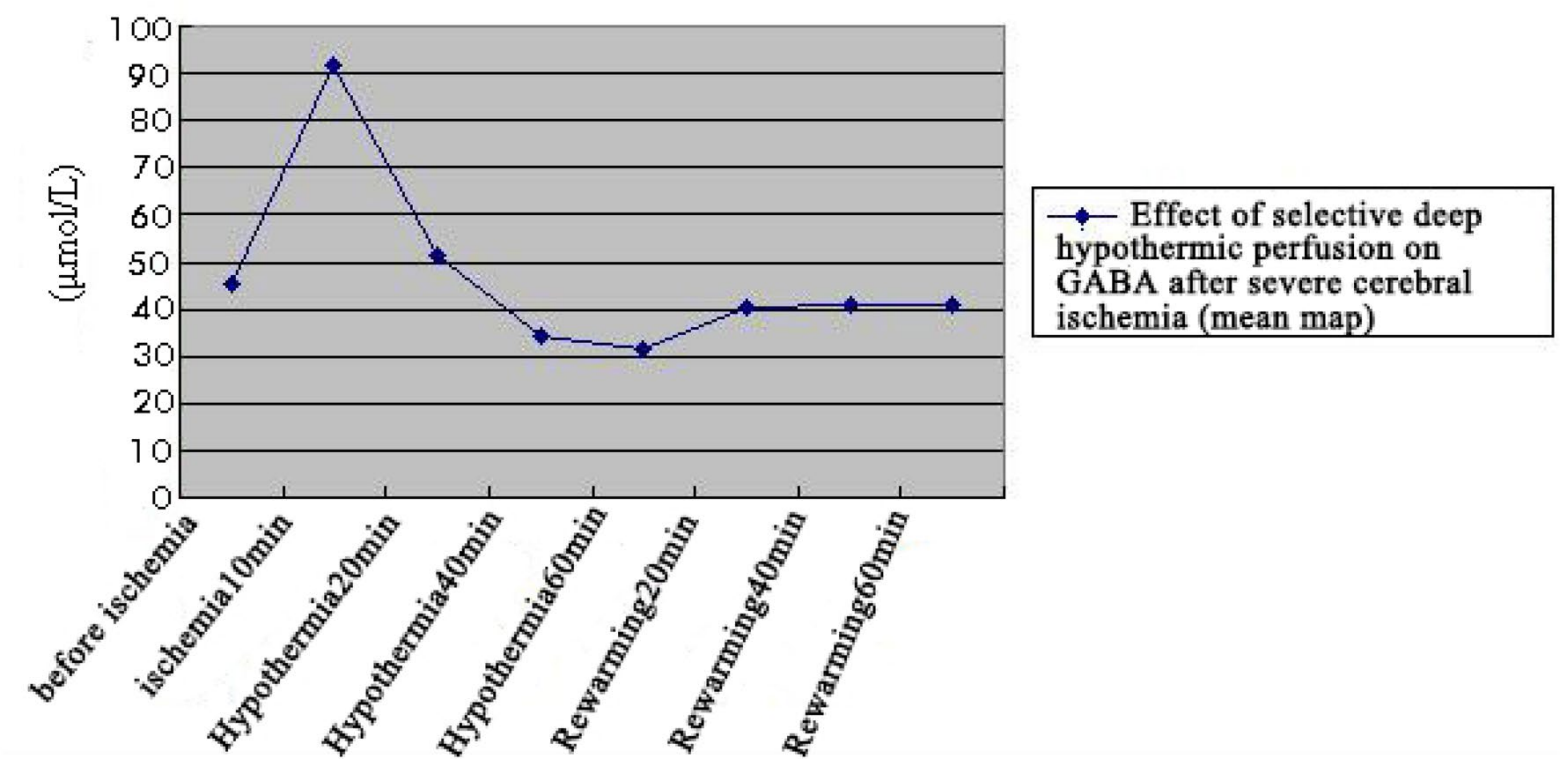
Changes of GABA content in brain extracellular fluid.

GABA concentration was determined in the extracellular fluid of brain tissue before 10 min of ischemia, after 10 min of ischemia, and during hypothermic perfusion and rewarming, and all quantitative data were expressed as mean ± standard deviation ($\overline{x}$ ± s) (Table 5). One-way ANOVA showed significant differences in GABA concentrations between different treatment groups (F = 351.74). There were significant differences in multiple comparisons between groups by the LSD method, and GABA concentrations in extracellular fluid reached the highest value at 10 min of ischemia (91.66 ± 4.01 µmol/L), which was significantly higher than those in the groups before ischemia, at different times of hypothermic perfusion and rewarming. The highest GABA concentration in ischemic fluid occurred at 10 min of ischemia (91.66 ± 1.01 µmol/L), which was significantly higher than that in the groups before ischemia, at different times of low-temperature perfusion and rewarming (Fig. 17).

**Table 5:** Changes in GABA content in brain extracellular fluid (μmol/L) ($\overline{x}$ ± s, n = 9).

Timing	GABA
Before ischemia	45.16 ± 3.38*^▲^
Ischemia for 10 min	91.66 ± 4.01
Hypothermic 20 min	51.54 ± 4.12*^▲^
Hypothermic 40 min	34.25 ± 2.78*^▲^
Hypothermic 60 min	31.44 ± 1.48*
Rewarmed 20 min	40.48 ± 2.11*^▲^
Rewarmed 40 min	40.72 ± 2.53*^▲^
Rewarmed 60 min	40.67 ± 3.02*^▲^
F	351.74
P	0.00

*Compared with ischemia for 10 min, *P < 0.05.

^▲^Compared with rewarmed 60 min, ^▲^P < 0.05.

Spearman correlation analysis of GABA concentrations with time groups in two temperature zones (cryogenic 20 min group, cryogenic 40 min group, cryogenic 60 min group, rewarming 20 min group, rewarming min group, and rewarming min clock group) depicted that the GABA content was significantly negatively correlated with cryogenic perfusion time (rs = −0.86**, P = 0.00) (Table 6), and not significantly positively correlated with rewarming time (rs = 0.20, P = 0.31) (Table 7).

**Table 6:** GABA values of brain extracellular fluid in different hypothermic perfusion time groups (μmol/L).

Timing	n	$\overline{x}$ ± s (μmol/L)	Rs	P
Hypothermic 20 min	9	51.54 ± 4.12	−0.86**	0.00
Hypothermic 40 min	9	34.25 ± 2.78		
Hypothermic 60 min	9	31.44 ± 1.48		

There was a significant correlation between different hypothermic perfusion times and GABA values.

**When the confidence (bilateral) is 0.01, the correlation is significant.

**Table 7:** GABA values of brain extracellular fluid in different rewarming time groups (μmol/L).

Timing	n	$\overline{x}$ ± s (μmol/L)	Rs	P
Rewarmed 20 min	9	40.48 ± 2.11	0.20	0.31
Rewarmed 40 min	9	40.72 ± 2.53		
Rewarmed 60 min	9	40.67 ± 3.02		

There was no a significant correlation between different rewarming times and GABA values.

**When the confidence (bilateral) is 0.01, the correlation is significant.

According to Mauchly sphericity test P < 0.10, the data do not conform to spherical data. There is a correlation between the results of multiple measurements, so ANOVA of repeated measurements is used, and the statistical results were corrected using Greenhouse–Geisser method. GABA repeated measurement ANOVA results of F = 85.044 after correction of the internal factor, P < 0.10, indicated that the overall means of GABA at different times were not the same, while the interaction time × temperature corrected F = 88.884, P < 0.10, indicated that there was an interaction between temperature and treatment time and that temperature had an effect on the measurements. The overall mean of GABA did not differ between the two temperature patterns F = 2.893 and P = 0.108 (Table 8).

**Table 8:** Analysis of variance of GABA repeated measures.

Variation	SS	df	Mean square	F	P
Time	1042.425	1.362	765.296	85.044	0.000
Temperature	32.335	1	32.335	2.893	0.108
Time × temperature	1089.497	1.362	799.854	88.884	0.000

After severe cerebral ischemia in rhesus monkeys, many amino acid neurotransmitters (Glu and GABA) were released from the extracellular fluid. The process of re-cold perfusion gradually decreased, and the decrease was significant compared to 10 min of ischemia, and the time of re-cold perfusion was significantly negatively correlated with the concentration of Glu and GABA, with both reaching the lowest value at 60 min of re-cold perfusion. Glu concentration gradually increased during the re-cold process and was significantly positively correlated with the time of re-cold. There was no significant correlation between the rewarming time and γ-aminobutyric acid. The change in Glu concentration had both temperature and time effects, and there was an interaction effect between temperature and time, whereas the change in GABA concentration was only affected by time, but there was an interaction effect between temperature and time.

## Discussion

Brain tissue has a very rich blood supply, and the blood flow accounts for about 20% of the whole body's blood supply. Therefore, brain tissue is very dependent on the blood supply and poorly tolerates ischemia and hypoxia. When the blood flow per 100 g of brain tissue drops to 40 mL/min, the brain tissue will suffer from ischemic-hypoxic damage. Complete interruption of blood supply to brain tissue for a few seconds at normothermia will lead to impaired consciousness for more than 5–6 min, and irreversible damage to brain tissue will occur^10,11^.

Neurotransmitters are inter-synaptic message-transmitting substances synthesized in neurons, released into the synaptic gap via presynaptic membrane terminals, and act on receptors on the postsynaptic membrane to produce postsynaptic potentials. Amino acid neurotransmitters are an important class of transmitters. Glutamic acid (Glu) and γ-aminobutyric acid (γ-aminobutyric acid) are, in turn, their main excitatory and inhibitory neurotransmitters, respectively^12^.

Glu is the main excitatory amino acid neurotransmitter in the central nervous system and is mainly found in the cerebral cortex and dorsal spinal cord^13^. Our findings are consistent with the increasing content of excitatory amino acid neurotransmitters in the extracellular fluid during cerebral ischemia, especially the content of Glu increases more significantly^14,15^. The large accumulation of Glu leads to a continuous depolarization of neurons, causing a positive feedback effect that leads to a significant increase in the release of Glu in neurons^13^. A series of chain reactions, including impaired cellular energy metabolism, toxic effects of excitatory amino acids, inflammatory responses, calcium overload, free radical responses, and cell death caused by cerebral ischemia and hypoxia, are central to brain damage caused by ischemia^16^.

GABA is mainly present in the cortex of the brain and cerebellum. Under physiological conditions, GABA is synthesized only in the brain^17^. Studies have shown that GABA exerts neuroprotective effects by forming pre-and postsynaptic inhibition in the synaptic gap, antagonizing glutamate depolarization, reducing cellular damage, and enhancing cellular tolerance to ischemia and hypoxia^18^. However, during persistent ischemic damage in the brain, the GABA content in the extracellular fluid is gradually reduced and depleted^17^, which is similar to our findings.

Olney et al.^14,15^ demonstrated that ischemia and hypoxia in brain tissue could stimulate a large release of the excitatory neurotransmitter Glu. Francisco et al.^19^ characterized the acute phase of cerebral ischemia by non-invasive magnetic resonance spectroscopy and observation of serum molecular markers of the inflammatory response under hypothermic, normothermic, and hyperthermic conditions in temperature-related glutamate excitotoxicity, brain tissue metabolic. Among the three damage mechanisms, glutamate excitotoxicity is the key molecular mechanism for the effect of temperature on the acute phase of cerebral ischemia. The present experimental study revealed by ANOVA with repeated measurements that there was an interactive effect between the changes in glutamate concentration during hypothermic perfusion and rewarming, not only with a temporal effect but also with a temperature factor, and the experimental results were consistent with the findings of Francisco et al.

Li et al. and Melani et al.^20–22^ found that GABA concentration increased significantly during ischemia, and with the release of excitatory amino acids, feedback caused an increase in GABA synthesis, antagonizing the "excitotoxic" effect of excitatory amino acids. In our study, GABA concentration increased significantly during ischemia, similar to the results of previous studies. The temperature factor was not significant in the process of GABA concentration change but only had a temporal effect.

Therefore, we found by repeated measurement ANOVA that in terms of neurotransmitter metabolism, hypothermic perfusion led to a decrease in the release of excitatory amino acids in neurons, reducing their "excitotoxic" effect and exerting a neuroprotective effect. Meanwhile, temperature changes had no significant effect on the metabolic changes of GABA, and the antagonistic effect of GABA on Glu during hypothermic perfusion was not significant. The antagonistic effect of GABA on Glu was not obvious during cryopreservation.

The neurotransmitters in the extracellular fluid were collected under anesthesia, and the effect of anesthetics on their metabolism could not be ruled out. Due to the limitation of microdialysis technology combined with high-performance liquid chromatography online analysis, the experimental results could only reflect the changes in amino acid neurotransmitter metabolism in different periods, represent the trend of their metabolic changes, and could not find the precise time inflection point of their content changes. The results can only be approximated as the trend of metabolic changes, and the precise temporal inflection point of the changes cannot be determined (Supplementary Information).

## Supplementary Information

Supplementary Information.

## Data Availability

The datasets used and analysed during the current study available from the corresponding author on reasonable request.
